# O Papel da Fibrose Atrial na Fibrilação Atrial: Nem Sempre Essencial?

**DOI:** 10.36660/abc.20230766

**Published:** 2023-12-19

**Authors:** Rodrigo Miguel-dos-Santos

**Affiliations:** 1 Smidt Heart Institute Cedars-Sinai Medical Center Los Angeles CA EUA Smidt Heart Institute, Cedars-Sinai Medical Center, Los Angeles, CA – EUA

**Keywords:** Arritmias Cardíacas, Fibrilação Atrial, Substrato Arritmogênico, Hipertensão Pulmonar

Estima-se que a arritmia cardíaca mais comum, a fibrilação atrial (FA), afete cerca de 2% da população mundial e espera-se que afete mais pessoas à medida que a população envelhece.^1–3^ Este cenário não é diferente no Brasil, onde se projeta que cerca de 2,5% da população seja afetada pela FA.^4,5^ A situação é mais alarmante entre os pacientes com hipertensão pulmonar, em que ∼30% podem apresentar episódios de FA e ∼70% de flutter atrial.^6,7^ Portanto, uma melhor compreensão dos mecanismos e substratos da FA é constantemente necessária para adequar melhor o tratamento dos pacientes.

Em publicação recente dos Arquivos Brasileiros de Cardiologia, Teixeira-Fonseca et al.,^8^ investigaram os efeitos das alterações morfofuncionais do átrio esquerdo induzidas pela hipertensão pulmonar na arritmogênese em átrios esquerdos isolados. A hipertensão pulmonar induzida pela monocrotalina levou à hipertrofia do átrio esquerdo, fibrose tecidual e remodelamento eletrofisiológico. Curiosamente, a estimulação elétrica *ex vivo* do tecido atrial esquerdo não provocou aumento de arritmias atriais no tecido de animais com hipertensão pulmonar. Conforme apontado pelos autores, alguns fatores podem ter contribuído para a ausência de arritmias *ex vivo*. Aqui, vou me concentrar na relação entre gatilho e substrato na FA e discutir a causa potencial para a falta de inducibilidade de arritmias.

Muitos fatores moleculares influenciam o desenvolvimento de arritmias atriais, mas, para acontecer, isso depende da interação de um gatilho de iniciação e de um substrato tecidual subjacente.^9^ A FA geralmente é iniciada por gatilhos originados nas veias pulmonares, que são causados por batimentos ectópicos ou disparos rápidos dessa área focal. Isso também é facilitado pela proximidade de um grande gânglio autonômico cardíaco, pela estrutura anatômica distinta da veia pulmonar e pelo perfil do canal iônico. No caso da hipertensão pulmonar, esse cenário é agravado pelas alterações da veia pulmonar causadas pela doença e pela elevação da pressão de enchimento do átrio esquerdo, que levam às alterações morfológicas observadas no átrio esquerdo dos pacientes com hipertensão pulmonar.^10^

O aparecimento de arritmias no miocárdio atrial também requer a presença de substratos arritmogênicos. Esses substratos cruciais são a remodelação do canal iônico, que perturba a estabilidade elétrica do tecido e facilita arritmias; inflamação, provocando a liberação de citocinas pelas células do sistema imunológico que também afetam a atividade elétrica atrial; e remodelação tecidual, que altera a arquitetura do miocárdio atrial e causa bloqueios de lentidão da condução. O mais preocupante é que as alterações causadas por estes substratos são agravadas por si mesmas. Embora todos esses substratos estejam presentes em pacientes com hipertensão pulmonar, maior foco tem sido dado à fibrose como substrato histológico crítico para FA devido à condução elétrica defeituosa e à predisposição à reentrada.^10,11^

Nos experimentos meticulosamente realizados em modelo de hipertensão pulmonar em ratos, Teixeira-Fonseca et al.,^8^ observaram a presença de fibrose atrial e alterações eletrofisiológicas. Ainda assim, a ausência de arritmias atriais quando um gatilho elétrico foi aplicado no átrio esquerdo pode nos levar a questionar o papel de outros fatores, como o sistema nervoso autônomo. Dada a necessidade de retirada do átrio esquerdo para o preparo *ex vivo*, a contribuição da inervação autônoma foi perdida. Então, ainda tem que ser explorada a potencial interação entre gatilhos, substratos e o sistema nervoso autônomo como modulador da FA no átrio esquerdo na hipertensão pulmonar.
