# Supplementary material for: O Papel da Fibrose Atrial na Fibrilação Atrial: Nem Sempre Essencial?
Source: Arq Bras Cardiol. 2023 Dec 19;120(10):e20230766. [Article in Portuguese] doi: 10.36660/abc.20230766 (PMC10773456; doi:10.36660/abc.20230766)
Supplement: Translation

# Translation: The Role of Atrial Fibrosis for Atrial Fibrillation: Not Always Essential?

**Authors:** Rodrigo Miguel-dos-Santos

**Affiliations:** 1 Los Angeles CA EUA Smidt Heart Institute, Cedars-Sinai Medical Center, Los Angeles, CA – EUA

**Keywords:** Arrhythmias, Cardiac, Atrial Fibrillation, Arrhythmogenic Substrates, Pulmonary Hypertension

The most common cardiac arrhythmia, atrial fibrillation (AF), is estimated to affect ∼2% of the worldwide population and is expected to impact more people as the population ages.^1–3^ This scenario is not different in Brazil, where ∼2.5% of the population is projected to be affected by AF.^4,5^ The situation is more alarming among pulmonary hypertension patients, in which ∼30% may present AF episodes and ∼70% atrial flutter.^6,7^ Hence, a better understanding of the mechanisms and substrates of AF is constantly needed in order to tailor patients’ treatment better.

In a recent publication of the *Arquivos Brasileiros de Cardiologia*, Teixeira-Fonseca et al.^8^ investigated the effects of the left atrial morphofunctional changes induced by pulmonary hypertension in the arrhythmogenesis in isolated left atria. Monocrotaline-induced pulmonary hypertension led to left atria hypertrophy, tissue fibrosis, and electrophysiological remodeling. Curiously, *ex vivo* electrical burst pacing of the left atrial tissue did not elicit increased atrial arrhythmias in the tissue from pulmonary hypertension animals. As pointed out by the authors, some factors may have contributed to the absence of arrhythmias *ex vivo*. Here, I am going to focus on the trigger and substrate relationship in AF and discuss the potential cause for the lack of arrhythmias inducibility.

Many molecular factors influence the development of atrial arrhythmias, but to happen, this is dependent on the interaction of an initiation trigger and an underlying tissue substrate.^9^ AF is usually initiated by triggers originating in the pulmonary veins, which is caused by ectopic beats or rapid firing from this focal driver area. This is also facilitated by the close proximity to a major cardiac autonomic ganglion, the distinct pulmonary vein anatomic structure, and its ion channel profile. In the case of pulmonary hypertension, this scenario is exacerbated by the changes in the pulmonary vein caused by the disease and the elevated left atrial filling pressure, which lead to the morphological changes seen in the left atrium of pulmonary hypertension patients.^10^

The onset of arrhythmias in the atrial myocardium also requires the presence of arrhythmogenic substrates. These crucial substrates are the ion channel remodeling, which disturbs the electrical stability of the tissue and facilitates arrhythmias; inflammation, provoking the release of cytokines by immune cells that also affect atrial electrical activity; and tissue remodeling, which alters the atrial myocardium architecture and cause conduction slowing blocks. Most concerning, the changes caused by these substrates are worsened by themselves. Although all these substrates are present in pulmonary hypertension patients, greater focus has been given to fibrosis as a critical histological substrate for AF due to the defective electrical conduction and predisposition to reentry.^10,11^

In the meticulously performed experiments in a rat model of pulmonary hypertension, Teixeira-Fonseca et al.^8^ observed the presence of atrial fibrosis and electrophysiological changes. Still, the lack of atrial arrhythmias when an electrical trigger was applied to the left atrium may lead us to wonder about the role of other factors, such as the autonomous nervous system. Given the need for the removal of the left atrium for the *ex vivo* preparation, the contribution of the autonomic innervation was missed. Then, it is still to be explored the potential interaction between triggers, substrates, and the autonomic nervous system as a modulator to AF in the left atrium in pulmonary hypertension.
